# Incorporating uncertainty in the baseline risk: An R Shiny tool and an empirical study

**DOI:** 10.1002/cesm.70018

**Published:** 2025-01-28

**Authors:** M. Hassan Murad, Lifeng Lin

**Affiliations:** ^1^ Evidence‐based Practice Center, Kern Center for the Science of Healthcare Delivery Mayo Clinic Rochester Minnesota USA; ^2^ Department of Epidemiology and Biostatistics University of Arizona Tucson Arizona USA

**Keywords:** baseline risk, GRADE, imprecision, meta‐analysis, Shiny App, uncertainty

## Abstract

The common practice in meta‐analysis and clinical practice guidelines is to derive the absolute treatment effect (also called risk difference, RD) from a combination of a pooled relative risk (RR) that resulted from a meta‐analysis, and a user‐provided baseline risk (BR). However, this method does not address the uncertainty in BR. We developed a web‐based R Shiny tool to perform simple microsimulation and incorporate uncertainty in BR into the precision of RD. We empirically evaluated this approach by estimating the impact of incorporating this uncertainty when BR is derived from the control group rates in 3,128 meta‐analyses curated from the Cochrane Library (26,964 individual studies). When BR was derived from the largest study in each meta‐analysis, the median width of the CI of BR was 11.6% (interquartile range (IQR), 6.30%–18.5%). Incorporating this uncertainty in BR led to expansion of the RD CI by a median of 8 per 1,000 persons (IQR 2–24). This expansion increased in a linear fashion with BR imprecision and was more prominent in meta‐analyses with low BR. This study provides a web‐based tool to perform simple microsimulation and incorporate uncertainty in BR into the CI of RD.

## 1 INTRODUCTION

In the context of meta‐analysis, clinical practice guidelines and decision modeling, a common practice is to derive the absolute treatment effect of binary outcomes from a combination of a pooled relative risk (RR) that resulted from a meta‐analysis, and a user‐provided baseline risk (BR) [1, 2]. The confidence interval (CI) of this absolute effect (also called risk difference, RD) is obtained following a substitution approach as proposed by Daly [3], in which the user‐provided BR is applied to the boundaries of the CI of RR to generate CI of the RD [1]. This is the default method implemented in Cochrane systematic reviews and in GRADEPro, the software that facilitates decision‐making following the GRADE approach (Grading of Recommendations, Assessment, Development and Evaluation) [1, 2]. However, an important limitation of this approach is that it does not account for the uncertainty in the BR [4, 5, 6]. Depending on the source of the BR, it may be derived from a small study and have a wide CI and important uncertainty, or it can be derived from a large study and be sufficiently precise. In addition, in some disease conditions, variability in BR may have more impact on variability in the outcome than the treatment itself.

Approaches to incorporate uncertainty in BR when estimating RD have been proposed [4, 5]. However, this analysis option is not available in dedicated meta‐analysis software, meta‐analysis packages in general statistical software, or in GRADEPro. Furthermore, the impact of incorporating uncertainty in BR on the imprecision of RD has not been empirically assessed. Knowing the magnitude of this increased uncertainty will be important for guideline developers because the RD CI is the basis for imprecision judgments and final grading of certainty. In this manuscript, we provide a web‐based R Shiny tool to help meta‐analysts address uncertainty in the BR. We test this approach on a very large sample of Cochrane systematic reviews to determine the average magnitude of uncertainty in BR and the impact of incorporating this uncertainty on the CI of the RD.

## 2 METHODS

### 2.1 The R Shiny tool

The tool allows users to choose a relative effect measure type, such as relative risk, odds ratio or hazard ratio, which needs to be provided with its CI. Users also need to enter a BR with its CI, or a range if CI is not available. The output of the tool includes the resultant RD (per 1,000 participants) and its CI based on both, the traditional approach (that does not incorporate BR uncertainty), and the new approach which incorporates BR uncertainty. Incorporating uncertainty in BR was done using microsimulation [4]. Microsimulation is a modelling method that simulates a set of data based on trajectories and characteristics of individual units [7]. In our specific context, a unit is an individual patient with a specific BR and RR. We sampled BR and RR from beta and lognormal distributions, respectively. The shape parameters of the beta distribution were obtained from the BR and its CI whereas the lognormal distribution parameters were obtained from the pooled RR and its precision [8]. Simulation was made with 10,000,000 draws. The tool is freely available online [9].

### 2.2 Empirical evaluation

This study used a convenience sample of meta‐analyses that were curated from the Cochrane Database of Systematic Reviews. Details of acquisition of this sample are provided elsewhere [10]. We excluded individual studies with zero events in both arms and meta‐analyses that included less than three studies. We only included one unique meta‐analysis from each systematic review, choosing the one with the largest number of included studies. If there were more than one such meta‐analysis, the first one presented in the review was chosen.

We used the control group event rate in the largest study in each meta‐analysis as a surrogate for BR in that meta‐analysis. We compared the expansion of the CI of the RD that takes place when uncertainty in BR is incorporated (defined as the difference between RD CI width before incorporating uncertainty and the width after incorporating uncertainty). The RD is rounded up to the next value and expressed per 1,000 patients treated (consistent with GRADE and Cochrane presentations). Analysis was done using the R software version 4.4.0 [11] with the meta package [12] and a published microsimulation code for RD estimation [4]. We report the median (50th percentile) values and interquartile ranges (IQR).

Sensitivity analyses were done based on three different RR meta‐analysis models: a random‐effects model using the DerSimmonian‐Laird method, a restricted maximum likelihood (REML) estimator of between‐study heterogeneity, and a common‐effect (fixed‐effect) model. We also conducted a sensitivity analysis in which BR was not derived from the largest study in each meta‐analysis, but rather by dividing the sum of events over the sum of sample sizes of the control groups in all included studies in a given meta‐analysis (the default approach in GRADEPro). The last analysis only included studies in which the control group received a placebo.

## 3 RESULTS

We included 3,128 meta‐analyses (26,964 individual studies, 20,390,937 participants).

### 3.1 Magnitude of uncertainty in BR

When BR was derived from the largest study in each meta‐analysis, it ranged from 0% to 100% (median 19.7%, interquartile range (IQR) 7.14%–41.4%). The width of the CI of BR had a median of 11.6% (IQR 6.30%–18.5%). When BR was derived from all the studies in a meta‐analysis, it ranged from 0.04% to 99% (median 21.5%, IQR 10.20%–39.5%). The width of CI of BR using this method was narrower than that calculated from the largest study in each meta‐analysis: 6.92% (IQR 4.03%–11.2%).

### 3.2 Expansion of the CI of RD

**Figure 1:**
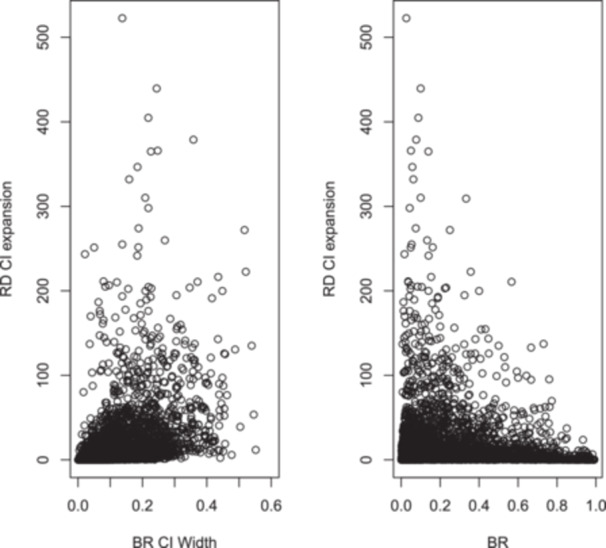
The Y axis reflects the expansion of the width of confidence interval of the risk difference (RD CI) when uncertainty in the baseline risk (BR) is incorporated in the RD CI. The unit of the Y axis is the number of events per 1,000 patients treated. The X axis represents the width of CI of BR (left panel) and the BR itself (right panel). The figure suggests more expansion of the RD CI as the CI of BR increases (left panel) and when BR value is low (right panel).

When uncertainty in BR was incorporated in the CI of the RD, the median expansion of the width of CI of the RD was 8 per 1,000 participants (IQR 2–24). The median and IQR using a REML estimator of heterogeneity, common‐effect model, placebo‐controlled trials and a BR derived from all the studies were 8 (2–24), 7 (2–23), 10 (3–31) and 3 (7–10), respectively. This RD CI expansion was stratified according to the magnitude of BR in Table 1. In Figure 1, we plot RD CI expansion against BR values and BR CI widths. The figure suggests that RD CI expansion was more prominent in studies with imprecise BR and in studies with low BR.

**Table 1:** Expansion of confidence interval of the risk difference when uncertainty in baseline risk is incorporated using microsimulation.

Baseline risk	Median expansion of confidence interval of the risk difference per 1,000 patients when uncertainty in baseline risk is incorporated				
	DerSimmonian Laird random‐effects model	Restricted maximum likelihood random‐effects model	Common‐effect model	Only placebo‐controlled studies	BR obtained from all the studies in a meta‐analysis
0–0.02	13	17	17	26	17
0.02–0.04	27	27	26	63	22
0.04–0.06	32	32	30	41	12
0.06–0.08	25	25	25	52	16
0.08–0.10	31	31	30	62	13
0.10–0.20	27	27	28	42	12
0.20–0.30	24	24	24	29	9
>0.30	8	15	15	18	7

## 4 DISCUSSION

This study provides additional rationale to incorporate uncertainty in BR when estimating absolute treatment effects and offers a methodology and a practical application for implementation. The magnitude of uncertainty in BR was not trivial, with a median width of the CI of BR of 12%. Incorporating this uncertainty increased the width of the CI of the RD by a median of 8–10 per 1,000 participants. This increase was more prominent when the control group was given placebo (vs other active or inactive comparators) and less prominent when the common‐effect model was used to generate the RR. The default approach in GRADEPro (deriving BR by dividing the sum of events by the sum of the sample sizes of the control groups) led to much less RD CI expansion suggesting that this approach likely underestimates the true precision of BR. This default approach may only be sensible when the heterogeneity in BR across studies is minimal. We also noted that RD CI expansion was more prominent in studies with low BR. Thus, clinical practice guidelines about conditions with low BR and rare outcomes have a stronger rationale to address uncertainty in BR to avoid a misleading impression of a precise RD.

This study has also shown the feasibility of using a simple microsimulation that is available in an open‐source code [4] to address uncertainty in BR, which we have implemented in a web‐based tool [10]. We encourage systematic reviewers and guideline developers to incorporate uncertainty in BR when estimating the absolute effects required for decision‐making. We also advocate for acknowledging heterogeneity in meta‐analysis by using different BRs for different population subgroups, each with its own precision, in order to obtain a fair and complete overview of the contextual impact of a treatment.

Several remaining limitations are important to highlight. To make this study feasible, we derived BR from studies that were included in the same meta‐analyses that generated the relative effect. This dependence can be avoided by using BR from an external source, which would be the preferred approach in practice [13] and is supported by the web‐based tool we have provided [9]. Aside from this dependence, the control group event rate itself is a poor surrogate of BR and is subject to various biases, such as measurement error [10]. Ideally, BR should be derived from a better source that maps to clear context‐specific prognostic risk factors. Furthermore, it is important to recognize that in addition to the statistical uncertainty in BR, there are other types of uncertainties that relate to non‐statistical factors (e.g., geographical or temporal). Both approaches, the traditional one and the proposed one which addresses uncertainty in BR, assume portability of the relative effect across various BRs. This assumption is not always true [10], and when lack of portability is suspected, models that produce conditional effect based on BR are needed [4]. The proposed approach has not been endorsed by the GRADE Working Group and will require evaluation in the context of future guidelines.

In conclusion, we encourage systematic reviewers and guideline developers to address uncertainty in BR when estimating absolute effects needed for decision‐making. We present one approach to conduct such analysis facilitated by an open‐source tool.

## AUTHOR CONTRIBUTIONS

**M. Hassan Murad**: Conceptualization, analysis, writing—original draft preparation. **Lifeng Lin**: Conceptualization; data curation; formal analysis; software; writing—review and editing.

## CONFLICT OF INTEREST STATEMENT

The authors declare no conflicts of interest.

## PEER REVIEW

The peer review history for this article is available at https://www.webofscience.com/api/gateway/wos/peer-review/10.1002/cesm.70018.

## Data Availability

Data are available from the Cochrane Library and can be provided by the corresponding author upon reasonable request.
